# Impact of an electronic clinical decision support system on workflow in antenatal care: the QUALMAT eCDSS in rural health care facilities in Ghana and Tanzania

**DOI:** 10.3402/gha.v8.25756

**Published:** 2015-01-27

**Authors:** Nathan Mensah, Felix Sukums, Timothy Awine, Andreas Meid, John Williams, Patricia Akweongo, Jens Kaltschmidt, Walter E. Haefeli, Antje Blank

**Affiliations:** 1 Department of Clinical Pharmacology and Pharmacoepidemiology, Heidelberg University Hospital, Heidelberg, Germany; 2 Navrongo Health Research Centre, Navrongo, Ghana; 3 Muhimbili University of Health and Allied Sciences (MUHAS), Directorate of Information and Communication Technology, Dar Es Salaam, Tanzania; 4 School of Public Health, University of Ghana, Legon Accra, Ghana

**Keywords:** electronic clinical decision support system, workflow, antenatal care, health care providers, sequence of events, rural setting, developing countries, sub-Saharan Africa

## Abstract

**Background:**

The implementation of new technology can interrupt established workflows in health care settings. The Quality of Maternal Care (QUALMAT) project has introduced an electronic clinical decision support system (eCDSS) for antenatal care (ANC) and delivery in rural primary health care facilities in Africa.

**Objective:**

This study was carried out to investigate the influence of the QUALMAT eCDSS on the workflow of health care workers in rural primary health care facilities in Ghana and Tanzania.

**Design:**

A direct observation, time-and-motion study on ANC processes was conducted using a structured data sheet with predefined major task categories. The duration and sequence of tasks performed during ANC visits were observed, and changes after the implementation of the eCDSS were analyzed.

**Results:**

In 24 QUALMAT study sites, 214 observations of ANC visits (144 in Ghana, 70 in Tanzania) were carried out at baseline and 148 observations (104 in Ghana, 44 in Tanzania) after the software was implemented in 12 of those sites. The median time spent combined for all centers in both countries to provide ANC at baseline was 6.5 min [interquartile range (IQR) =4.0–10.6]. Although the time spent on ANC increased in Tanzania and Ghana after the eCDSS implementation as compared to baseline, overall there was no significant increase in time used for ANC activities (0.51 min, *p*=0.06 in Ghana; and 0.54 min, *p*=0.26 in Tanzania) as compared to the control sites without the eCDSS. The percentage of medical history taking in women who had subsequent examinations increased after eCDSS implementation from 58.2% (39/67) to 95.3% (61/64) *p*<0.001 in Ghana but not in Tanzania [from 65.4% (17/26) to 71.4% (15/21) *p*=0.70].

**Conclusions:**

The QUALMAT eCDSS does not increase the time needed for ANC but partly streamlined workflow at sites in Ghana, showing the potential of such a system to influence quality of care positively.

Health information technology (health IT) improves the quality of care and patient safety in the health sector (1, 2). Developed countries have successfully used health IT to improve aspects of health care such as medical and medication errors and adverse drug events (2–4) . Electronic health records have been used increasingly to manage and document the care process in these countries. Often, they build the backbone for connected intelligent health IT solutions such as algorithm-driven, interactive electronic clinical decision support systems (eCDSSs). Because these systems have mostly been implemented in large urban and academic hospitals in developed countries, the experience and knowledge gained from their use mainly relate to these settings (5, 6).

In resource-poor settings, the implementation of health IT is difficult (7–10) , and barriers for successful implementation are often underestimated (11–13) . The deployment of health IT in rural resource-poor settings has been limited so far by lack of finances, adequate IT infrastructure, and trained staff (9). Common characteristics like understaffing, fears of frequent downtimes due to vulnerable infrastructure, and the fear of a potential increase in workload influence health care providers’ attitudes and decrease the adoption of such systems (7, 8, 14). However, eCDSSs have been successfully used in rural settings to deal with the lack of trained clinical personnel, reduce workload in understaffed institutions, and issue reminders and alerts to optimize HIV therapy (7, 15–17) .

Although eCDSS technology appears promising, its initial deployment almost inevitably influences the workflow of users, thus requiring workflow adjustments with the potential to increase workload (18, 19). Misalignment between a proposed electronic system and an established workflow may create workarounds bypassing the computer system, and this can have unknown consequences (18, 20). In their review, Kawamoto and coworkers identified that one of the critical success factors of an eCDSS is *the integration of an eCDSS with charting or the order entry system to support workflow integration* (21). It is therefore of utmost importance to analyze the workflow of a target setting for an eCDSS prior to the software development and after implementation. The most appropriate method – although there are only very few reports of such studies in resource-poor settings – is to use time and motion techniques to analyze the influence of an eCDSS on the workflow (17, 22).

This study was conducted in Ghana and Tanzania as a sub-study of the Quality of Maternal and Prenatal Care: Bridging the Know-Do Gap (QUALMAT) research project. The QUALMAT project was funded as part of the 7th Framework Programme of the European Union (grant agreement 22982) and was a collaboration between the Centre de Recherche en Santé de Nouna (Burkina Faso), Ghent University (Belgium), Heidelberg University (Germany), Karolinska Institutet (Sweden), Muhimbili University of Health and Allied Sciences (Tanzania), and Navrongo Health Research Centre (Ghana). Each southern partner country included a policy advisory board with local experts from national and subnational authorities. The board members were involved in the planning of the project and its studies and the discussion of results. The overall objective of QUALMAT was to improve the motivation and performance of health workers and ultimately the quality of prenatal and maternal care services at rural primary health care facilities. The intervention packages included the development and implementation of an eCDSS based on World Health Organization (WHO) guidelines and the introduction of performance-based incentives (23). All interventions were evaluated in a controlled pre-post study design in rural Burkina Faso, Ghana, and Tanzania between 2009 and 2014 (23, 24).

The objective of this study was to evaluate the workflow of health care providers before and after the implementation of the QUALMAT eCDSS, specifically to evaluate the order of major task categories, and the time spent on major task categories during antenatal care (ANC) in rural primary health care facilities in Ghana and Tanzania. We hypothesized that using the eCDSS would increase the time required to provide ANC. However, we also hypothesized that the system would increase guideline adherence and streamline the order of activities.

## Methods

### Study design

The study was a one-sequence, two-phase (before and after), parallel group intervention using an observational time–motion assessment focusing on the major task categories performed by health care providers. The pre-implementation survey was carried out between October and December 2011, and the post-implementation survey was carried out 17 months after implementation of the final version of the eCDSS (September to November 2013).

The study was conducted at 12 intervention and 12 non-intervention health care facilities, designated as QUALMAT study sites, equally distributed between two resource-limited districts in Ghana (Kassena-Nankana and the adjoining Builsa district) and two districts in Tanzania (Lindi Rural and Mtwara Rural); that is, in each country, 12 health care facilities participated. The population sizes of the Kassena-Nankana and Builsa districts are 153,000 and 95,800, respectively, whereas those of Lindi rural and Mtwara rural districts are 216,000 and 205,000, respectively. All study districts were remote, rural, and (within their respective countries) comparable in terms of relevant medical infrastructure, equipment, and staff. In the two districts in Ghana, each district has a district hospital located within a 30–45 km radius from the health care facilities that serves as the referral facility. Similarly, in Tanzania, the district referral hospitals are located within a radius of 50–100 km away from the health care facilities. Burkina Faso was also part of the main QUALMAT project; however, due to divergent timelines and competing research priorities in the participating countries, sites in Burkina Faso were not part of this sub-study.

Ethical approval for this study was obtained from the Ethics Committee of the Medical Faculty of Heidelberg University, Germany (S-173/2008); the Institutional Review Board of the Navrongo Health Research Centre, Ghana (ID: NHRCIRB116); and the Muhimbili University of Health and Allied Sciences Ethical Review Committee, Tanzania (Ref. No. MU/RP/AEC/Vo. XIII). Written informed consent of observed health care providers was sought and verbal consent was obtained from all pregnant women whose interaction with care providers was observed. Permission was also obtained from the regional and district health authorities to carry out the study in their health care facilities.

### Observation setting and eCDSS implementation

The workflow of randomly selected health care providers involved in the provision of ANC services at the study sites was observed during the study. In Ghana, providers were midwives and community health nurses with 2 to 3 years of professional training. In Tanzania, all staff members in the facilities were involved in the provision of maternal and neonatal care and had varying professional training ranging from 1 to 4 years. The ANC services were mainly provided by nurses and medical attendants (auxiliary staff), as described by Prytherch and colleagues (25). Study sites were similar in relevant aspects of provision of care; however, the organization of ANC services varied across the health care facilities. In Ghana, providers were exclusively assigned to the maternal care unit without rotation to other health care departments. The client registration, the vital signs assessment, and the dispensing of preventive drugs to pregnant women were usually carried out in a hallway by community health nurses. The midwife or other qualified staff provided the remaining elements of the ANC service in a separate room. In some health care facilities, in addition to the ANC services, staff members performed basic screening tests for syphilis, HIV, and hepatitis using bedside diagnostic test kits. However, for malaria and more laborious diagnostic tests, patients were sent to laboratories located nearby.

In Tanzania, focused ANC services were provided in a designated ANC room. The providers were rotated on a shift basis within the facility hence no permanent staff were assigned to ANC services. The client registration was done in the hallway by auxiliary staff or a nurse. When more than one staff member was involved in the provision of care, the tasks were split. The main ANC consultation and the physical examination were carried out in the ANC room. Staffing at sites is detailed in Supplementary Table 1

**Table 1:** Distribution of observations (n), proportion (%), and time (min) spent on major task categories at baseline in intervention and non-intervention sites in Ghana and Tanzania

		All ANC (214)		First ANC (*n*=70)				Follow-up ANC (*n*=144)				
					
	Major task categories	Observations (*n*)	Time (%)^a^	Observations (*n*)	Time (%)^a^	Mean time (±SD) (min)	Median (min)	Observations (*n*)	Time (%)^a^	Mean time (±SD) (min)	Median (min)	*p*
1	Welcome	50	1.8	11	1.0	1.33 (±0.65)	1.72	39	2.5	0.88 (±0.6)	1.00	0.05
2	Registration	94	6.3	58	9.1	2.20 (±2.11)	1.54	36	3.4	1.30 (±0.79)	1.00	0.12
3	History taking	129	12.1	53	14.6	3.85 (±7.71)	2.63	76	9.5	1.71 (±6.99)	0.66	<0.001
4	Physical examination	142	8.8	55	10.3	2.62 (±1.88)	1.00	87	7.3	1.14 (±0.81)	1.00	0.116
5	Vital sign	164	12.0	59	9.3	2.19 (±2.39)	2.00	105	14.7	1.92 (±1.93)	1.45	0.26
6	Obstetric examination	197	10.3	64	6.6	1.45 (±1.14)	1.00	133	14.0	1.43 (±1.10)	1.05	0.99
7	Laboratory investigation	57	14.2	35	23.6	9.45 (±12.49)	5.67	22	4.6	2.83 (±2.43)	2.00	0.001
8	Urinalysis	10	0.7	8	1.0	1.75 (±1.17)	2.00	2	0.4	3.00 (±1.41)	3.00	nd
9	Drug administration	170	12.8	58	8.3	2.01 (±2.14)	1.23	112	17.3	2.11 (±2.47)	1.16	0.86
10	Client education	181	21.1	56	16.1	4.02 (±3.53)	2.55	125	26.2	2.87 (±2.99)	2.00	0.02

a Total does not add up to 100% due to rounding; nd=not done due to low occurrences.

The eCDSS was developed to provide guidance and decision support at the point of care (23). The software was used during patient care. Implementation started by a presentation of the software to the district's management. This was followed by basic computer and QUALMAT eCDSS software training for health care providers at the intervention sites. In Ghana, each trainee received a total of 12 days of training, whereas in Tanzania, 11 days of training were provided, with each training session lasting 5 hours on average (26). New care providers joining the team were also included in the refresher training. A technical support team visited each health care facility biweekly to solve problems and answer questions. A telephone hotline was installed to allow technical support to be contacted when immediate assistance was needed.

### Observation procedure and data collection

Observation of workflow was carried out in both the intervention and non-intervention districts about 6 months before and 17 months after eCDSS launch. Observers used a stopwatch and took notes on a standardized data collection sheet that predefined the major task categories and activities to be witnessed (27). Observation involved the provision of a detailed descriptive account of activities and the sequence in which they occurred (28). The method was adapted from the assessment of medication handling errors (29, 30) and from other workflow studies (31, 32).

Observers were trained for 4 hours on how to identify ANC processes and record the start and end times of major task categories as predefined on data collection sheets. In Ghana, a trained research assistant and a researcher (NM) conducted the observations, whereas in the 12 sites in Tanzania, two experienced midwives conducted the observations. The observers visited the health care facilities between 8:00 am and 3.30 pm, which were the official working hours, and average observations lasted about 4 hours. They aimed to observe as many first ANC visits as possible because they were more laborious, differed in important aspects from later visits, and were less frequent. The observers were stationed so as not to obstruct the health care providers, but were close enough to hear and see what the health care providers were saying and doing. To ensure the privacy of clients, the observers stayed behind the screens during physical examinations and during counseling on sensitive issues. To minimize interference with care and to enable simultaneous observation in different locations within the ANC clinic, one person was stationed in the examining room and another in the hallway. If patients moved between places, the observation sheet was passed on to the researcher inside the respective examination room to complete the observation process and to keep track of the sequence. The observer identified individual task categories performed by the health care provider and recorded the starting time when the interaction with the pregnant woman began. The task ended when the staff member switched to another major task. All task categories that were carried out inside the ANC clinic were observed, whereas those performed outside of the ANC unit could not be followed. Hence, if the midwife referred the pregnant women to the laboratory for other investigations, we were unable to observe this task. Staff interviews were conducted to validate the observations.

Workflow was defined as the sequence of activities or operations performed by various ANC providers using available resources over time (33, 34). Any visit of a pregnant woman to the health care facility to seek ANC service other than the first ANC visit was considered a follow-up ANC visit. Observation was defined as watching a pregnant woman going through services provided. Time was defined as time spent by the health care provider to provide the service. A time-and-motion assessment was used to quantify the time that health care providers spent on ANC activities (17, 35). Six of the 10 major task categories of the ANC process were predefined on the basis of the WHO guidelines: 1) welcome, 2) registration, 3) history taking, 4) physical examination, 5) vital signs, and 6) obstetric examinations. The remaining are 7) laboratory investigations, 8) urinalysis, 9) drug administration, and 10) client education (Supplementary Table 2) (36).

**Table 2:** Distribution of observations (n), proportion (%), and time (minutes) spent on major task categories before and after implementation of the eCDSS in Ghana

		Before eCDSS implementation (*N*=75)				After eCDSS implementation (*N*=64)				
				
	Major task categories	Observations (*n*)	Time (%)	Mean time (±SD) (min)	Median time (min)	Observations (*n*)	Time (%)	Mean time (±SD) (min)	Median time (min)	*p*
1	Welcome	8	0.48	0.42 (±0.67)	0.20	3	0.03	0.12 (±0.03)	0.13	0.54
2	Registration	22	6.70	2.12 (±1.91)	1.32	32	11.88	5.18 (±2.93)	4.58	<0.001
3	History taking	38	7.56	1.38 (±1.70)	0.59	55	15.69	3.98 (±4.47)	1.83	<0.001
4	Physical examination	39	6.30	1.12 (±0.94)	1.00	64	12.11	2.64 (±2.70)	2.03	<0.001
5	Vital sign	45	15.49	2.39 (±2.39)	1.85	64	9.67	2.12 (±1.04)	1.99	0.62
6	Obstetric examination	60	13.09	1.51 (±1.28)	1.21	64	6.94	1.51 (±0.70)	1.37	0.12
7	Laboratory investigation	14	13.79	6.85 (±7.81)	4.88	52	22.84	6.12 (±5.12)	6.26	0.82
8	Urinalysis	0		–	–	0	–	–	–	–
9	Drug administration	52	16.09	2.15 (±2.66)	1.10	52	8.85	2.37 (±1.56)	2.13	0.004
10	Client education	60	20.49	2.37 (±2.95)	1.08	64	12.00	2.61 (±1.78)	2.20	0.004

### Data analysis

The data were checked, entered, and validated using EpiData 3.0. The data were analyzed using Stata 11.0 (StataCorp, College Station, TX, USA) and the R software/environment version 3.1.0 (R Foundation for Statistical Computing, Vienna, Austria). The time spent was computed as a difference between the ‘end-time’ and ‘start-time’ variables while subtracting potential interruptions. Descriptive statistics were used to summarize the data by means of arithmetic means, standard deviations (SD), median, and interquartile ranges (IQR) depending on the distribution. Between-group differences comparing intervention and non-interventions sites or before-and-after intervention were assessed using the non-parametric Mann–Whitney test. Investigating the time attributable to the implementation of the eCDSS, we used a linear model including the categorical variable for phase (before or after eCDSS implementation), the categorical variable for group (intervention or non-intervention), and the interaction between them, if necessary. We also analyzed the order in which history taking and the combination of physical and obstetric examinations were performed. All tests were two-tailed, 95% confidence intervals (CI) were calculated, and *p*<0.05 was considered statistically significant. Graphical display was carried out using GraphPad Prism 5.01 (GraphPad Software Inc., USA).

## Results

### Major task categories of ANC over time at sites in Ghana and Tanzania

On average, 1.6 midwives/nurses and 2 other auxiliary staff members provided services at each health care facility in Ghana, whereas 1.6 midwives/nurses and 4 other auxiliary staff provided the services in Tanzania (Supplementary Table 1). Of the 108 (40 in Ghana/68 in Tanzania) health care providers who took part in the initial study, 58 (53.7%; 19 in Ghana/39 in Tanzania) remained at their site until the end of the study. At baseline 214 observations (144 in Ghana/70 in Tanzania) and after implementation of the eCDSS 148 observations (104 in Ghana/44 in Tanzania) were carried out in the 24 QUALMAT health care facilities. Hence, within the 8-week data collection period, 8.9 observations per site (range 5–14) were conducted. Seventy (33%) were first ANC visits, and the remaining were follow-up ANC visits.

The baseline data for the intervention and non-intervention sites in both countries showed no statistical difference. They were combined to describe the baseline workflow at the ANC clinic as detailed in Table 1. Obstetric examination was the most frequently conducted task category occurring in 92.1% (197/214) of all visits. This was followed by client education in 84.6% (181/214) and drug administration in 79.4% (170/214), as shown in Table 1. Laboratory investigation was one of the least frequently observed task categories and accounted for 26.6% (57/214) of all visits. When comparing the proportion of time spent on each task category, client education accounted for 21.1% of the total contact time, followed by laboratory investigations (14.2%), and drug administration (12.8%, Table 1). Before implementation of the eCDSS, the median time spent to provide ANC services to clients was 6.5 min (IQR=4.0–10.6) for Ghana and Tanzania combined. The median duration of first ANC visits was 10.3 min (IQR=6.8–19.3), and follow-up ANC visits lasted 5.2 min (IQR=3.7–8.9). Laboratory investigations took the longest time (5.67 min, IQR=4.0–8.9) and accounted for 23.6% of the total contact time in the first ANC visits group.

For data collected after the implementation of the eCDSS in six sites in each country, analyses were grouped by countries and data from intervention sites were compared with those from non-intervention sites. The time spent on task categories at the intervention sites significantly increased in Ghana as compared to the baseline data of the respective sites. The average duration of an ANC visit more than doubled from 7.4 min (IQR=2.9–9.5) before to 19.2 min (IQR=9.0–28.2) after the eCDSS implementation (*p*<0.001). But there was also a 49% increase in time observed at the non-intervention sites from 8.5 min (IQR=4.5–9.0) to 12.7 min (IQR=6.2–16.5) after 17 months, which, however, did not reach significance (*p*=0.09). The tasks requiring more time after the eCDSS implementation in Ghana were registration (3.26 min, *p*<0.001), history taking (1.24 min, *p*<0.001), physical examination (1.03 min, *p*<0.001), drug administration (1.03 min, *p*=0.004), and client education (1.12 min, *p*=0.004), which corresponds to an approximately twofold increase (Table 2).

In Tanzania, the average duration of an ANC visit as compared to baseline increased significantly by 69% from 15.1 min (IQR=9.0–17.8) to 25.5 min (IQR=16.4–33.9) in the intervention site (*p*<0.001) and contrasted sharply with the non-significant 120% increase in the non-intervention sites from 8.8 min (IQR=5.0–9.0) to 18.3 min (IQR=8.0–22.5, *p*=0.06) after 17 months. The tasks that took more time after eCDSS implementation in Tanzania were registration (4.18 min, *p*<0.001), vital signs (2.02 min, *p*<0.001), obstetric examination (1.17 min, *p*<0.002), and laboratory investigations (6.72 min, *p*<0.004), as shown in Table 3.

**Table 3:** Distribution of observation (n), proportion (%), and time (minutes) spent on major task categories before and after implementation of the eCDSS in Tanzania

		Before eCDSS implementation (*N*=34)				After eCDSS implementation (*N*=32)				
				
	Major task categories	Observations (*n*)	Time (%)	Mean time (±SD) (min)	Median time (min)	Observations (*n*)	Time (%)	Mean time (±SD) (min)	Median time (min)	*p*
1	Welcome	11	1.63	1.00 (±0.00)	1.00	27	3.07	1.06 (±0.83)	0.93	0.18
2	Registration	26	6.68	1.73 (±2.38)	1.00	25	18.07	6.72 (±3.82)	5.18	<0.001
3	History taking	24	8.01	2.25 (±2.17)	2.00	16	2.87	1.67 (±1.71)	1.02	0.09
4	Physical examination	30	8.16	1.83 (±1.05)	2.00	23	4.90	1.98 (±1.64)	1.20	0.64
5	Vital sign	34	11.28	2.24 (±3.14)	1.00	32	11.03	3.21 (±1.67)	3.02	0.001
6	Obstetric examination	34	6.83	1.35 (±1.23)	1.00	32	9.46	2.75 (±1.89)	2.17	0.002
7	Laboratory investigation	22	16.62	5.09 (±6.12)	2.00	26	27.76	9.93 (±7.56)	8.72	0.004
8	Urinalysis	10	2.67	2.25 (±1.16)	2.00	0	–	–	–	–
9	Drug administration	30	13.50	3.03 (±3.52)	2.00	28	10.53	3.50 (±3.60)	1.96	1.0
10	Client education	33	24.62	5.03 (±4.05)	3.00	27	12.31	4.24 (±2.89)	4.32	0.60

### Major task categories of ANC over time assessed between intervention sites with eCDSS and non-intervention sites

The overall effect of the eCDSS, adjusted for baseline values, when comparing the intervention with the non-intervention sites, did not show any change in time spent in Ghana (0.51 min, *p*=0.06) and Tanzania (0.54 min, *p*=0.26). When comparing individual tasks in the intervention versus the non-intervention sites in Ghana, the task on which significantly more time was spent in the intervention site was the physical examination. There was a trending toward increasing time spent on registration. Conversely, there was a trend toward decreasing time spent on laboratory investigations and obstetric examinations (Table 4). Similarly, in Tanzania, obstetric examinations showed a significant increase in time. However, the time spent on registration only showed an increasing trend. Conversely, the time spent on history taking, physical examination, and client education showed a decreasing time trend (Table 4).

**Table 4:** Change in time spent after 17 months comparing intervention (with eCDSS) versus non-intervention sites in Ghana and Tanzania

	Intervention vs. non-intervention (Ghana)				Intervention vs. non-intervention (Tanzania)			
		
Major task categories	Change in time/(min)^a^	SE (min)	*p*	95% CI (min)	Change in time/(min)^a^	SE (min)	*p*	95% CI (min)
Welcome	–	–	–	–	0.07	0.30	0.82	(−0.51; 0.63)
Registration	2.08	1.15	0.07	(0.20; 4.36)	1.41	1.43	0.33	(−1.43; 4.25)
History taking	1.00	2.31	0.67	(−3.57; 5.56)	−3.98	3.99	0.32	(−11.97; 4.01)
Physical examination	1.30	0.54	0.02	(0.23; 2.36)	−1.44	2.84	0.61	(−7.07; 4.19)
Vital sign	0.42	0.41	0.31	(−0.40; 1.23)	0.57	0.88	0.52	(−1.18; 2.32)
Obstetric examination	−2.03	1.10	0.07	(−4.21; 0.14)	2.14	0.56	<0.001	(1.03; 3.26)
Laboratory investigation	−4.70	3.17	0.14	(−11.01; 1.60)	2.64	5.92	0.66	(−9.19; 14.46)
Urinalysis	–	–	–	–	–	–	–	–
Drug administration	−0.01	0.53	0.99	(−1.05; 1.03)	−1.12	1.29	0.39	(−3.67; 1.43)
Client education	0.35	0.68	0.61	(−0.99; 1.69)	−2.14	1.43	0.14	(−4.97; 0.69)

a Change in time (minutes) comparing intervention and non-intervention sites using ANOVA.

### Frequency and order of performing task categories

**Fig. 1:**
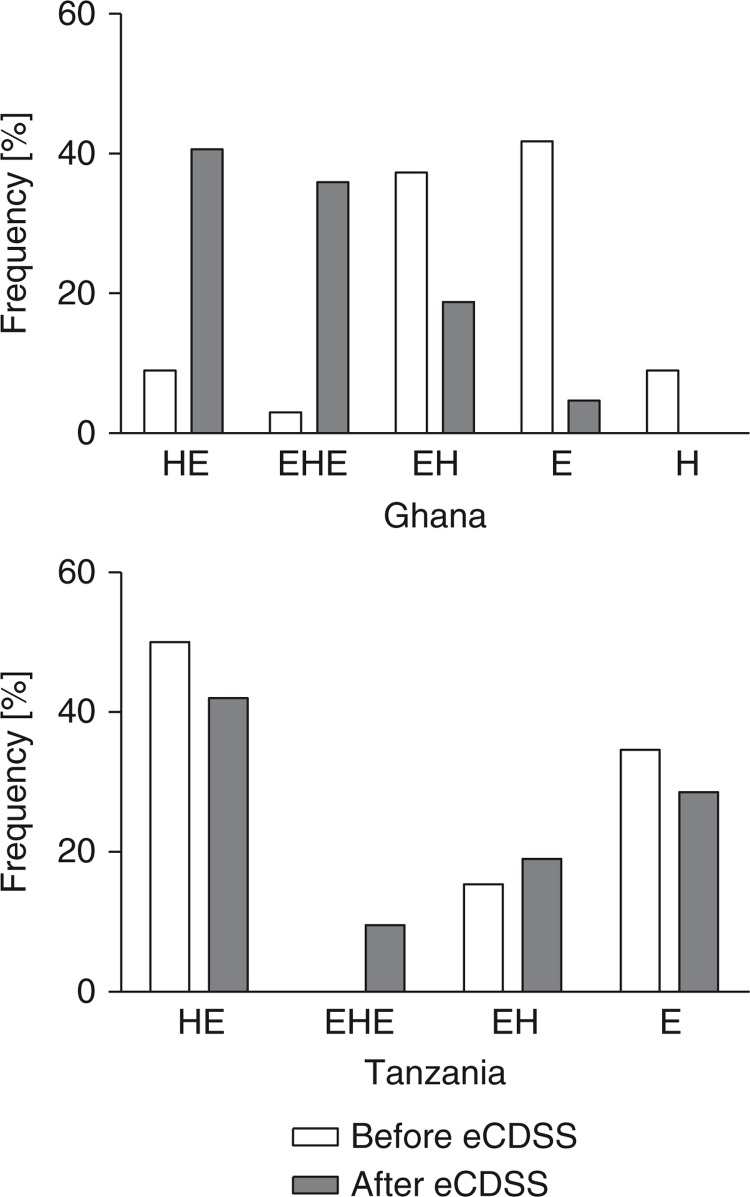
Sequence of performing history (H) and examination (E) before and after implementation of the eCDSS in Ghana and Tanzania.

The three key tasks of history taking, physical examination, and obstetric examination did not show a strict sequential order of performance at baseline, and often one was performed without the other. Because physical and obstetric examinations are very closely related tasks that cannot always be clearly separated, we will refer subsequently to physical and/or obstetric examinations as simply *examinations*. After eCDSS implementation, fewer examinations were conducted without a prior history [before, 41.8% (28/67); after, 4.7% (3/64); *p*<0.001] in Ghana, whereas no change was observed in Tanzania [34.6% (9/26) vs. 28.6% (6/21), *p*=0.64] (Fig. 1). The percentage of examinations conducted without a prior history was reduced from 37.3% (25/67) to 18.8% (12/64, *p*=0.02) in Ghana and remained unchanged in Tanzania [15.4% (4/26) vs. 19.0% (4/21), *p*=0.73]. The percentage of history taking conducted between examinations increased from 3.0% (2/67) to 35.9% (23/64, *p*<0.001) in Ghana and showed an increasing trend from 0.0% (0/26) to 9.5% (2/21, *p*=0.07) in Tanzania, likely representing a higher total frequency of history taking. There were fewer occurrences of history taking conducted without physical examination and vice versa. For example, history taking without examinations was not observed after the eCDSS implementation in Ghana (Fig. 1). Overall, the percentage of medical history taking in women who had subsequent examinations increased after eCDSS implementation from 58.2% (39/67) to 95.3% (61/64, *p*<0.001) in Ghana and remained the same in Tanzania [65.4% (17/26) vs. 71.4% (15/21), *p*=0.70].

## Discussion

Our study investigated whether and how the implementation of the QUALMAT eCDSS influenced the various tasks performed during ANC care. The most important finding is that the QUALMAT eCDSS did not increase the duration of the ANC process at the study sites in Ghana and Tanzania as compared to the non-intervention sites. This was an unexpected finding because both staff and members of the study team had expressed fears about increasing time for ANC care by using the eCDSS.

Increased time spent after implementation of the eCDSS may have occurred because health care providers used the eCDSS in parallel to their normal, mandatory, paper-based system. Unfortunately, this situation cannot be avoided in pilot projects like QUALMAT. The complete replacement of paperwork by an electronic system would need a broad national consent, which is not achievable for only six study sites in a district. The fact that the time spent on ANC also increased at the non-intervention sites, however, confirmed that the time increase triggered by the eCDSS did not substantially differ from normal fluctuation and underscores the importance of having a control group.

The quest for quality improvement in health care results in countless national and non-governmental approaches to change care in rural settings in Ghana and Tanzania. As an example, in Ghana an independent project aimed at strengthening health systems, especially in the area of maternal and newborn health, was concurrently conducted in the non-intervention sites while our observations were being undertaken (37). This may well explain why quality also changed in the control sites. Hence, a true and conclusive assessment of the effect of the QUALMAT eCDSS (and other interventions in such a setting) is possible only by drawing comparisons with the sites without the electronic support. This applies even more for the assessment of changes in quality of care at the QUALMAT sites, which is still ongoing.

In this controlled setting, we showed that the use of the QUALMAT eCDSS did not increase the time spent for ANC compared to similar sites without an eCDSS in Ghana and Tanzania. We suggest that where manual paper entry could be reduced further in scaling up the electronic system, the care process may become more efficient. This has been shown in an electronic medical record system, which was uniformly used within one hospital (10). Often, clinicians are concerned that new technology may require more time to carry out standard activities (38). Our findings can help to overcome these fears and support the adoption of similar new electronic systems (12, 39).

In addition, the eCDSS has the potential to change the mode and the frequency of performing tasks. We showed a positive influence on the percentage of history taking at the sites in Ghana, where history taking increased significantly after the eCDSS implementation, and in addition the order of performing task categories was positively influenced. While there are recommendations on the task activities for the provision of ANC services by the WHO and national authorities, there is no emphasis on the order in which ANC tasks and activities should be performed. However, for many activities, there is a medically meaningful order, and the omission of activities may always raise questions of quality and safety. As an example, a current patient history is an indispensable source of information that may focus and guide physical examination. The positive influence of the eCDSS implementation in Ghana on history taking and examinations is promising and may lead to better quality of care. However, interestingly, this effect was not seen in Tanzania. One reason for this may have been that the sites in Tanzania had a higher number of staff working with the eCDSS and a higher turnover of staff during the lifetime of the QUALMAT study. One consequence of the high turnover of staff may therefore have been that users were on average less familiar with the system, compared to their colleagues in Ghana.

Vital signs are very basic indicators, and elevated blood pressure is an early initial sign of a condition rather than a life-threatening situation. However, our system was not able to increase the frequency of vital signs assessment. For an eCDSS, any omission of activities (input) may result in a possible failure of the automated algorithm-based detection of relevant changes. It is challenging to balance mandatory entries and minimal interference with the process and workload. The QUALMAT system chose to guide caregivers through the ANC process, but it did not ask for mandatory data entry except in respect to patient identification. This approach was chosen because the target users were not experienced in handling computers, and mandatory data entry may cause frustration and dangerously detract users from the care process. On the other hand, omissions can have various reasons, and if vital signs are not taken, this may also be due to missing or defective equipment. Previous studies in similar settings showed that shortages of skilled staff, equipment, and consumables accounted for some ANC procedures not being carried out in Burkina Faso and Tanzania (40, 41). Since it is impossible to predict how the system will affect health care delivery during the development phase, achieving a balance between mandating activities and minimal interference should be given careful consideration during the development of an eCDSS. Future systems may give consideration to adopting a stricter approach in this regard.

Evidence-based medicine broadly supports health care; however, the challenge still remains of getting health care providers to accept and practice it (39). Current health IT policies in Ghana and Tanzania are changing, and health IT is becoming an integral part of health education and health care delivery in resource-poor countries. Electronic tools may support the focus on evidence-based medicine, and the QUALMAT project could show that health care personnel are waiting for health IT. Challenges to implement such a system may be overcome, and the costs may be justifiable [Sukums F personal communication, Saronga HP personal communication, and (26, 42)]. However, the study presented here showed both expected and unexpected results and added to what was previously known in this area (2, 3, 17, 20). This emphasizes the need to conduct other time and motion studies in a variety of settings prior to systems being scaled up for routine patient care.

### Study strength and limitations

Interestingly, the findings in Ghana and Tanzania, in West Africa and East Africa respectively, were broadly comparable. The controlled setting increased the credibility of the data and allowed one to define the true impact of the eCDSS at the sites. The rural settings in northern Ghana and southern Tanzania represent a typical resource-poor African setting nested in two different health systems. The strength of our findings therefore is that the comparability of the data between the two countries encourages the findings to be applied to other resource-poor rural health care sites as well.

Some limitations of our study also warrant discussion. The number of observations carried out in each site was not uniform and was lower than we originally planned. We know that this potentially underpowered the result of the study. We were, however, confined to the QUALMAT study sites and could not increase the number of (intervention) sites for observations.

A second limitation in the study was our inability to use the same observers in both countries. This was not only due to financial constraints but also due to the difficulty in finding people fluent in the different local languages. However, we believe that having had the same observers throughout the study likely minimized bias. Therefore, this situation did not affect the results too much. Another limitation was that, although an observer was stationed in the examination room in an unobtrusive way, the possibility of the Hawthorne effect cannot be ruled out (27). Both health care providers and their clients were aware of being watched. This might have influenced the duration of the procedures, presumably making them longer. However, this influence should have been equal in both study arms. Finally, the organization and ANC setup in some facilities made observations difficult. As an example, observations of laboratory investigations reflected only those conducted within the ANC premises, as we could not follow women referred to the main laboratory, which was often located outside the ANC units. Finally, although time and motion studies quantify the amount of time spent on performing tasks, they do not provide information on the quality of care. The quality-of-care assessment at the QUALMAT study sites is still ongoing, and the success of the eCDSS as a whole can be truly assessed only after it is known if there were any expected or unexpected changes in quality of care.

## Conclusion

The use of the QUALMAT eCDSS for ANC did not increase the time spent on ANC visits. At study sites in Ghana, more task categories were performed in a better organized way, and the percentage of history taking increased significantly. We therefore conclude that the QUALMAT eCDSS in part streamlined the workflow, underscoring the potential to increase the medical effectiveness of ANC processes.

## Authors' contributions

NM, WEH, and AB designed the study, conducted data analysis, and were responsible for interpretation of data and writing of the manuscript. NM implemented and conducted the study in Ghana. FS participated in the design of the study and implemented and conducted the study in Tanzania. TA and AM supported the statistical analysis. TA, AM, JW, PA, and JK participated in data interpretation and the manuscript writing. All authors substantially contributed to this article.

## Supplementary Material

Impact of an electronic clinical decision support system on workflow in antenatal care: the QUALMAT eCDSS in rural health care facilities in Ghana and Tanzania
