# Supplementary material for: Impact of an electronic clinical decision support system on workflow in antenatal care: the QUALMAT eCDSS in rural health care facilities in Ghana and Tanzania
Source: Glob Health Action. 2015 Jan 27;8:10.3402/gha.v8.25756. doi: 10.3402/gha.v8.25756 (PMC4309829; doi:10.3402/gha.v8.25756)
Supplement: Impact of an electronic clinical decision support system on workflow in antenatal care: the QUALMAT eCDSS in rural health care facilities in Ghana and Tanzania [file GHA-8-25756-s001.pdf]

Supplementary Material

Impact of an electronic clinical decision support system on workflow in antenatal care: The QUALMAT eCDSS in rural health care facilities in Ghana and Tanzania

Nathan Mensah[1,2], Felix Sukums[1,3], Timothy Awine[2], Andreas Meid[1], John Williams[2], Patricia Akweongo[4], Jens Kaltschmidt[1], Walter E. Haefeli[1], Antje Blank[1]*

Supplementary Table 1: Staffing at the 24 study health care facilities in Ghana and Tanzania

Health facility	Ghana				Tanzania			
	Midwives	Auxiliary staff	Laboratory staff	Total staff	Nurses**	Auxiliary staff*	Laboratory staff	Total staff
**1**	2	0	yes	2	3	5	No	8
**2**	1	4	yes	5	3	4	yes	7
**3**	2	0	yes	2	1	6	No	7
**4**	2	3	yes	5	1	5	Yes	6
**5**	1	3	yes	4	2	3	Yes	5
**6**	2	0	yes	2	2	5	No	7
**7**	2	4	yes	6	1	5	No	6
**8**	1	0	yes	1	2	3	No	5
**9**	1	3	no	4	3	4	No	7
**10**	1	2	yes	3	0	3	Yes	3
**11**	1	2	yes	3	0	1	Yes	1
**12**	1	2	yes	3	2	4	No	6

* In Tanzania auxiliary staff comprised medical attendants, health assistants, clinical officers, and assistant medical officer.

** In Tanzania: Enrolled nurses and registered nurses acted as midwives. In Ghana auxiliary staff comprised community health nurses.

Supplementary Table 2: Definition of major task categories of ANC care

Major task category		Description of ANC activities
1	Welcome	Welcoming the client to the ANC facility by the health care provider
2	Registration	Involves issuing the ANC registration and serial number, asking and documenting basic personal information of the client into her ANC booklet and register.
3	History taking	Involves asking and documenting past obstetric history, lactation history of last child, medical/surgical history, family history of illnesses, menstrual history, history of contraceptive use, and allergies to drug and food in the ANC booklet.<br><br>Also addresses current complaints related to pregnancy.
4	Vital signs taking	Taking and documenting height, weight, blood pressure, pulse, and temperature of the client in the ANC booklet and register.
5	Physical examination	This involves general examination from head to toe, pallor, looking for signs of edema and abdominal scars, and the examination of breast and pelvis.
6	Obstetric examination	Palpation of abdomen, measuring of fundal height, listening of fetal heart sound, checking fetal position presentation, checking and documenting gestational age.
7	Laboratory investigation	Taking of blood samples and testing for syphilis and HIV using rapid diagnostic test kits in the ANC clinic. Testing for malaria parasites, hemoglobin, and checking blood grouping of client.
8	Urinalysis	Testing of urine for protein, glucose, and acetone.
9	Drug administration	This includes vaccination (tetanus) and dispensing preventive drugs such as antimalaria tablet (sulfadoxine-pyrimethamine) for pregnant women between 16-36 weeks; anthelminthics (mebendazole), folic acid, and iron folate supplement tablets.

10	Health education and counseling	Educating of the client on topics such as: danger signs in pregnancy, use of insecticide impregnated materials, diet, nutrition, hygiene, need for immunization, STI prevention, Voluntary counseling and testing, mother to child transmission of HIV, safer sex, family planning, birth preparedness; and readiness for complicated delivery

HIV- human immunodeficiency virus; STI-sexually transmitted infections
